# Slow progress in diarrhea case management in low and middle income countries: evidence from cross-sectional national surveys, 1985–2012

**DOI:** 10.1186/s12887-017-0836-6

**Published:** 2017-03-21

**Authors:** Chandrashekhar T. Sreeramareddy, Yue-Peng Low, Birger Carl Forsberg

**Affiliations:** 1 Department of Community Medicine, International Medical University, Bukit Jalil, Kuala Lumpur Malaysia; 2 Institute of Biological Sciences, Faculty of Science, University of Malaya, Kuala Lumpur, Malaysia; 3 Health Systems and Policy Research (HSP), Department of Public Health Sciences, Karolinska Institutet, S-17177 Stockholm, Sweden

**Keywords:** Under-5 child, Diarrhea, Oral rehydration therapy, Case management, Child health services, Developing countries, Trends

## Abstract

**Background:**

Diarrhea remains to be a main cause of childhood mortality. Diarrhea case management indicators reflect the effectiveness of child survival interventions. We aimed to assess time trends and country-wise changes in diarrhea case management indicators among under-5 children in low-and-middle-income countries.

**Methods:**

We analyzed aggregate data from Demographic and Health Surveys and Multiple Indicator Cluster Surveys done from 1986 to 2012 in low-and-middle-income countries. Two-week prevalence rates of diarrhea, caregiver’s care seeking behavior and three case management indicators were analyzed. We assessed overall time trends across the countries using panel data analyses and country-level changes between two sequential surveys.

**Results:**

Overall, yearly increase in case management indicators ranged from 1 · 3 to 2 · 5%. In the year 2012, <50% of the children were given correct treatment (received oral rehydration and increased fluids) for diarrhea. Annually, an estimated 300 to 350 million children were not given oral rehydration solutions, or recommended home fluids or ‘increased fluids’ and 304 million children not taken to a healthcare provider during an episode of diarrhea. Overall, care seeking for diarrhea, increased from pre-2000 to post-2000, i.e. from 35 to 45%; oral rehydration rates increased by about 7% but the rate of ‘*increased fluids’* decreased by 14%. Country-level trends showed that care seeking had decreased in 15 countries but increased in 33 countries. Care seeking from a healthcare provider increased by ≥10% in about 23 countries. Oral rehydration rates had increased by ≥10% in 15 countries and in 30 countries oral rehydration rates increased by <10%.

**Conclusions:**

Very limited progress has been made in the case management of childhood diarrhea. A better understanding of caregiver’s care seeking behavior and health care provider’s case management practices is needed to improve diarrhea case management in low- and-middle-income countries.

## Background

Globally, an estimated 5.8 million deaths in children less than 5 years old occurred in the world in 2015 [1]. The millennium development goal four (MDG-4) was not met in 28 countries, 11 of which had a under-5 mortality in excess of 100 per 1000 livebirths. Experts argue that that MDG-4 could not be accomplished since childhood diarrhea which accounts for about 10% of all under-5 child deaths [2] was not effectively tackled owing to inadequate implementation of existing evidence-based interventions for diarrhea [3, 4]. Though a 60% reduction of child mortality rate from diarrhea has been achieved between 2000 and 2013, a further reduction targeted during the sustainable development goals (SDG) era requires a further improvement in coverage and quality of diarrhea case management [5].

In 1978, the World Health Organization (WHO) recommended that Oral Rehydration Salts (ORS) or Recommended Home Fluids (RHF) be used for all acute watery diarrheas among children to prevent deaths from diarrhea dehydration [6] leading to the establishment of diarrheal diseases control programs with technical support from United Nations Children’s Fund (UNICEF) [7] and WHO member states [8]. Later, WHO-UNICEF joint strategy aimed to improve the proportion of children with diarrhea who receive ORS and increased fluids to 80% by 2000 [9]. Case management of diarrhea under Integrated Management of Childhood Illness strategy (IMCI) [10] by training the healthcare providers (HCP) has been shown to improve their case management skills [11].

Scaling up the existing evidence-based interventions may avert most of the diarrheal deaths [12–14]. Yet, only a third of under-5 children with diarrhea are given an appropriate and prompt healthcare [15]. Therefore, a Global Action Plan for Pneumonia and Diarrhea (GAPPD) [16] has underscored the importance of studying caregivers’ knowledge about symptoms, recognition of danger signs, healthcare seeking behavior and identifying the barriers of access to [17]. Estimating total burden of under-5 children incorrectly managed for diarrhea would help prioritizing disease-specific interventions to further reduce under-5 mortality, and policy making and advocacy control of childhood diarrhoea.

In many low- and middle-income countries (LMICs), healthcare seeking behavior and caregivers’ recognition of childhood illnesses is inadequate and use of oral rehydration therapy (ORT) for diarrhea is unacceptably low [18]. The reasons are not clearly understood due to the complexity of caregivers’ decision making process [19]. To close the gap between the burden of childhood illnesses and appropriate healthcare given to the sick children, 52 countdown countries have adopted community case management (CCM) strategies [20] and methods for monitoring trends of community-based treatment coverage for childhood illnesses using households surveys are being explored [21]. We aimed to assess the trends of diarrhea case management indicators in LMICs from national-level household surveys to better understand the impact of IMCI scale-up on those indicators.

## Methods

### Data sources and procedures

The conceptual framework for this updated analyses was adapted from a report on diarrhea case management by Forsberg et al.[18]. In this report, we included data on case management indicators on childhood diarrhea up to the year 2012. We used aggregate data from Demographic and Health Surveys (DHS) [22] and Multiple Indicator Cluster Surveys (MICS) [23] done in LMICs between the years 1985 and 2012 for which data were available during July, 2014. The data were obtained from http://www.statcompiler.com/for DHS and http://www.micscompiler.org/for MICS. The aggregate data on 2-week prevalence of diarrheal episode, case management indictors were extracted from both data sources and merged into a single file. DHS is a national level, household survey on representative samples of men and/women conducted by the DHS program. MICS is a national or sub national survey conducted on a sample of women in the reproductive age group. DHS are supported by United States Agency for International Development while MICS are supported by the UNICEF. Briefly, in both DHS and MICS, women aged 15–49 years are interviewed about reproductive health, child health and nutrition. Both surveys are conducted at regular intervals of about 5 years. The use of core standardized and comparable questionnaires allows for cross-country comparisons and time-trend analyses.

In both DHS and MICS, women are first asked to list all the children aged 0–59 months. For each child listed, mothers were asked about episodes of diarrhea during 2 weeks prior to the date of interview. If diarrhoa had occurred, they were asked about treatment measures taken and healthcare seeking behavior during the episode/s of diarrhea. From the information gathered following indicators were constructed for monitoring of diarrhea case management.

1. prevalence of diarrhea;
2. percentage of children diarrhea who were taken to a healthcare provider (any type);
3. percentage of children having diarrhea who were given ORS alone;
4. percentage of children having diarrhea who were given either ORS or RHF;
5. percentage of children having diarrhea who were given increased fluids (IF) compared with their regular intake.

Among the indicators listed above, indicator 1 quantifies the burden of diarrheal diseases and indicator 2 describes the care seeking behavior of caregivers during diarrhea. Indicators 3 to 5 measure immediate management of childhood diarrhea by use or ORS and/or RHF, and increased fluids to correct and/or prevent dehydration. Indicators for all the countries and years for which data were available were included in our analyses.

### Statistical analyses

We estimated the predicted values for all available data for indicators 2–5, by panel data analyses using *‘xtset’* command on Stata 10.1. At first, for each variable, a country code was created as a panel variable, and year of survey was included as time variable to perform random-effects generalized linear regression analyses. The yearly trend was presented as trend lines. The coefficients from linear regressions were used to estimate annual change in case management indicators. For those countries in which two or more surveys were done between 2000 and 2012, or if the interval between previous two surveys was at least 10 years, we calculated the absolute differences in diarrhea case management indicators to assess country-level changes between two sequential surveys.

For each country and each survey (year-wise), we used the prevalence of diarrhea and each specific indicator (use rate as %) reported in the survey, to estimate the total number of children reported of having diarrhea and the total number of children who were taken to health care provider (HCP), given ORS or RHF in a particular year for each country. For these estimations, we substituted the 2-week prevalence rates and diarrhea case management indicators in under-5 population data for the particular country and the year. Under-5 population data was obtained from the International Database of the United States Census Bureau [24]. From the estimated numbers, for each survey, we computed 2-week prevalence rates and diarrhea case management indicators by summing up the total number of children with diarrhea, taking the proportion who were taken to HCPs and received treatments and dividing them by the total under-5 population and total under-5 children with diarrhea respectively.

## Results

### Data characteristics

Data on various indicators were available for a total of 71–116 countries from one or more surveys in each country (from either DHS and/or MICS), during the time period from 1985 to 2012 and the total number of surveys ranged from 71 to 282 in all the countries (data not shown), depending on the case management indicator that was assessed.

In total, data about diarrhea case management was available from 285 surveys (77 countries). The numbers for the total surveys and countries included in this analysis varied since the number of DHS and MICS done in each country differed and data on some indicators was not available in some surveys. Data on childhood diarrhea was available from DHS and MICS for a total of 914.1million under-5 children (Table 1).

**Table 1:** Two-week prevalence of diarrhea episodes among under-5 children, the proportion of children taken to a Health Care Provider (HCP), and utilization rates of Oral Rehydration Solutions (ORS) either ORS or Recommended Home Solutions (RHS), rates of giving increased fluids during diarrhea in 195 DHS and MICS surveys 1986–2000 and 2001-2012

Year of survey	Number of surveys	Populations of under-5 children	Diarrhea episodes	% of children taken to HCP	ORS usage	Use of ORS/RHS	Increased fluids
1986	5	27,056,419	19 · 9	NA	12 · 9	22 · 5	NA
1987	8	20,618,700	22 · 2	NA	8 · 2	8 · 9	NA
1988	6	*9,533,465*	23 · 5	NA	17 · 1	19	NA
1989	3	4,182,865	12 · 8	NA	20 · 7	62 · 6	NA
1990	5	22,620,086	15 · 4	37 · 1	25 · 6	34 · 6	10 · 5
1991	7	45,705,697	12 · 6	41 · 5	38 · 7	50 · 9	39 · 8
1992	9	21,113,642	18 · 6	29 · 2	24 · 7	34 · 1	33 · 7
1993	3	21,334,523	15 · 6	30 · 3	19 · 8	29	42 · 7
1994	2	22,963,445	12 · 9	48 · 9	43	43 · 6	53 · 6
1995	3	6,095,295	17 · 9	31	36 · 6	40 · 6	47 · 3
1996	7	27,899,694	13	32 · 2	42 · 2	50	55 · 2
1997	4	46,640,427	12 · 6	46 · 6	41 · 2	43 · 2	55 · 1
1998	8	29,937,289	16 · 2	38 · 7	28 · 2	39 · 1	57 · 3
1999	6	12,882,673	9 · 2	20 · 9	48 · 2	60	45 · 6
2000	13	57,690,557	16 · 9	25 · 4	22 · 8	29 · 3	33 · 6
**Subtotal (1986–2000)**	**89**	**376,274,777**	**15 · 2**	**34 · 8**	**28 · 1**	**35 · 7**	**41**
2001	5	7,818,054	18 · 7	24 · 1	32 · 4	37 · 3	37 · 5
2002	6	7,553,275	12	48 · 8	35 · 3	46 · 4	32 · 5
2003	9	76,981,766	15 · 7	26 · 1	25 · 8	35 · 5	33 · 2
2004	8	44,646,303	12 · 1	29 · 4	46 · 3	50 · 8	44 · 8
2005	14	39,093,839	46 · 1	48 · 9	26 · 3	29 · 4	14 · 5
2006	9	69,319,312	20 · 1	57 · 3	37 · 1	42 · 9	22 · 9
2007	9	52,867,885	12 · 5	47 · 4	47 · 4	56 · 2	36 · 9
2008	10	71,737,289	11 · 1	45	39	44 · 7	22 · 4
2009	5	3,272,996	13 · 7	44 · 8	35 · 8	41 · 9	47 · 7
2010	11	29,317,712	14 · 2	46 · 3	39 · 4	48 · 6	31
2011	10	53,685,090	12 · 3	40 · 6	37 · 9	42 · 5	25 · 6
2012	10	81,551,555	17 · 6	59 · 1	38 · 6	44 · 1	23 · 7
**Subtotal (2001–2012)**	**106**	537,845,076	**16 · 8**	**45 · 4**	**35 · 2**	**41 · 5**	**26 · 1**
**Total (1986–2012)**	**195**	914119853	**16 · 1**	**41 · 6**	**32 · 2**	**39**	**31 · 5**

NA-data was not available

The bold text indicates the totals for the time periods

### Trends in case management of diarrhea

The number of surveys in each year, the overall population of under-5 children surveyed, and diarrhea case management indicators are shown in Table 1. Based on the current case management indicators from DHS and MICS data during the first decade of the 21st century the total numbers (in millions) of children suffering from diarrhea who were not given ORS, ORS-RHF and *‘increased fluids’* were 304.4, 348.5, 314.7, and 397.5 millions, respectively. Further 304.4 million children were not taken to a HCP. Though these estimates are only indicators of the magnitude of the problem of sub-optimal diarrhea management, the numbers may help mobilization of resources at global and national levels to improve childhood diarrhea case management. Overall use rates for ORS, ORS-RHF were <50%. From pre-2000 to post-2000 period, the indicator ‘*increased fluids*’ decreased from 41 to 31.5% and there was a marginal increase in ORS use rate (28.1 to 32.2%) and ORS-RHF rate (35.7 to 41.5%).

**Fig. 1:**
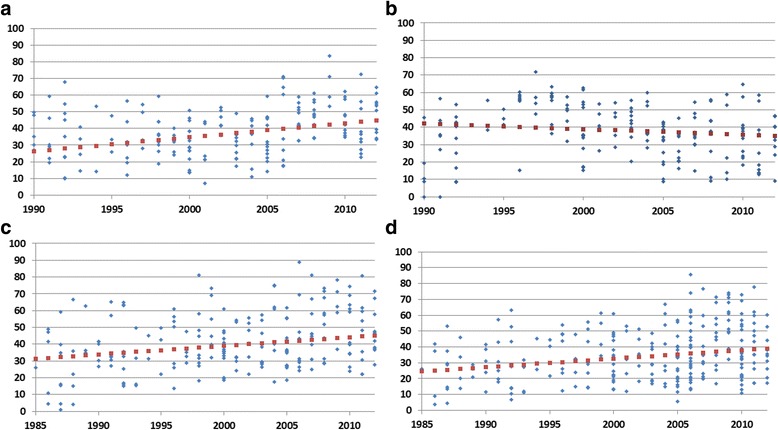
**a** The estimated average trend of the proportion of children taken to a health care provider during episodes of diarrhea in 201 surveys (DHS and MICS) between 1990 and 2012. **b** The estimated average trend for the proportion of children given ORS during an episode of diarrhea in 282 surveys (DHS and MICS) between 1985 and 2012. **c** The estimated average trend for the proportion of children given either ORS or RHS during an episode of diarrhea in 223 surveys (DHS and MICS) between 1985 and 2012. **d** The estimated average trend for the proportion of children given *‘increased fluids’* during an episode of diarrhea in 200 surveys (DHS and MICS) between 1990 and 2012

**Fig. 2:**
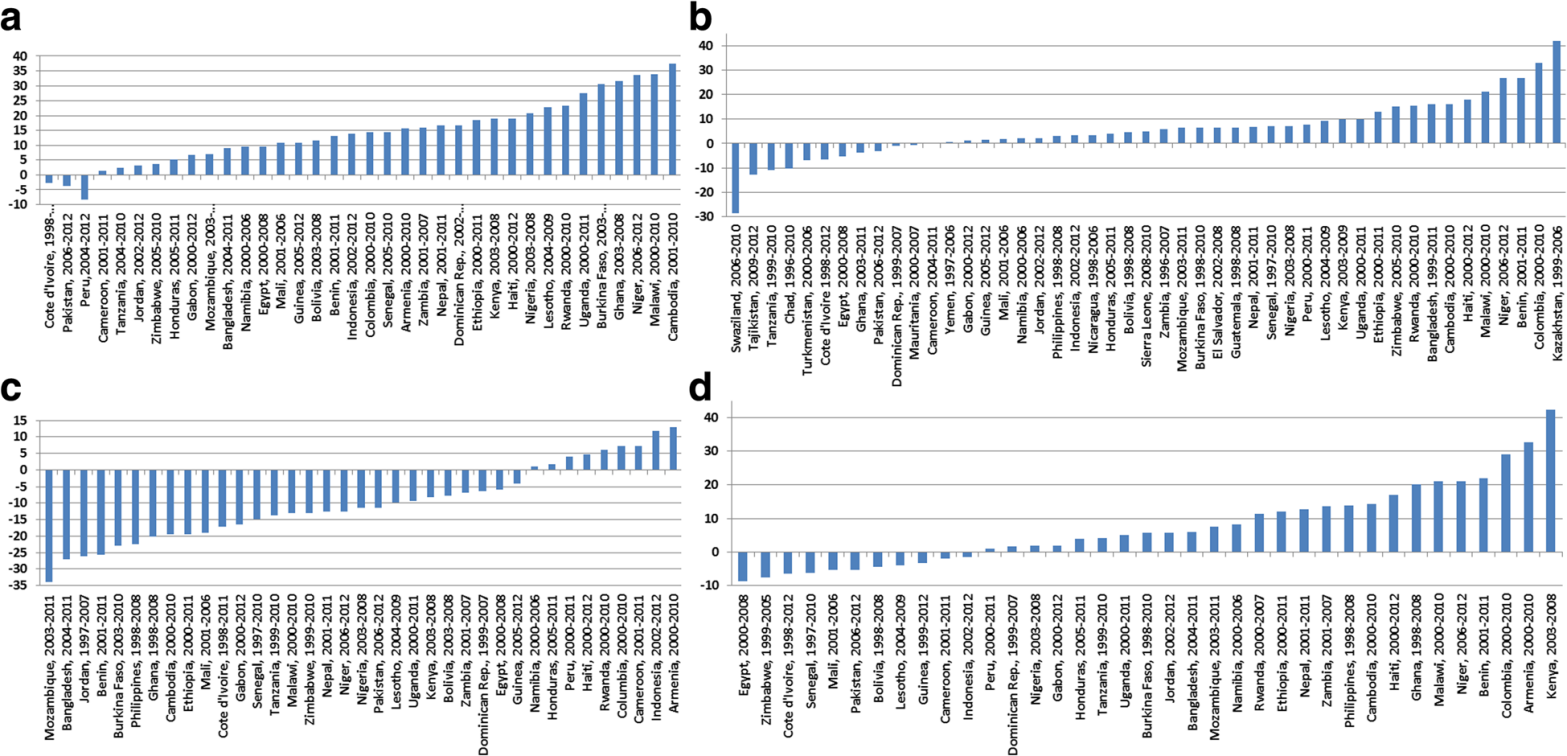
**a** Change in proportion of children taken to a Health Care Provider (HCP) for diarrhea in 36 countries 1998–2012. **b** Change in proportion of children given ORS for diarrhea in 47 countries 1996–2012. **c** Change in proportion of children given either ORS or Recommended Home Fluids (RHF) for diarrhea in 37 countries 1998–2012. **d** Change in proportion of caretakers stating they gave their children more fluids to drink than usual during diarrhea in 37 countries 1997–2012

The time-trend analyses for annual rates of change showed that most case management indicators had increased by only 1.3 to 2.5% (Fig. 1a-c) except for ‘*increased fluids’,* which showed a yearly decline of 1.1% (*p* = 0.039) (Fig. 1d). Yearly increments for the proportion of children taken to a HCP for diarrhea was 2.5% (*p* < 0.001), for ORS use rate was 1.7% (*p* < 0.001) and for ORS-RHF use rate was 1.3% (*p* < 0.001). Country-level change in diarrhea case management indicators between two sequential surveys are shown in Fig. 2a to d.

The proportion of children taken to a HCP during diarrhea was only 41%. From pre-2000 to post-2000 period the proportion of children taken to a HCP for diarrhea increased from 34.8 to 45.4% and had increased in 34 of the 37 countries for which data were available. In 23 of them the rate increased by ≥10% while it decreased in only 3 countries. Increase in consulting a HCP was highest in Cambodia (37.8%) followed by Malawi (33.8%) (Fig. 2a).

Data on country-level change in ORS use rate between two sequential surveys was available for 47 countries. Of these, 36 countries showed an increase in ORS use rates up to 10% only while 11 countries showed a decrease in ORS use rate.. Swaziland had experienced 28.5% decreased ORS use rate while higher increases were seen in Kazakhstan (42%) and Columbia (32.9%) (Fig. 2b). Data on ORS-RHF use rates were available for 37 countries of which 11 countries experienced a decreasing (<10%) ORS-RHF use rate. Among the remaining countries, ORS-RHF rates had increased by ≥10% in 15 countries; Kenya (42.3%), and Armenia (32.6%) being the highest (Fig. 2c). Data on country-level change in ‘*increased fluids’* was available for 38 countries, of which in 20 countries ‘the rate decreased by >10% while it increased in only 9 countries (by 13% in Armenia and 11.7% in Indonesia). Large declines in ‘*increase fluid*s’ rates were found in Mozambique (33.9%), and Bangladesh (27.1%), (Fig. 2d).

## Discussion

Our key findings were that a very little progress had been made in case management of diarrhea in LMICs over the period 1985–2012. Our data suggests that seeking treatment from a HCP as well as use of ORS and/or RHF for childhood diarrhea increased slightly over the period while rates of *‘increased fluids’* during childhood diarrhea had decreased. None of the case management indicators had reached anywhere near the target of 90% set by the global community [25]. Rates for use of ORS, ORS-RHF and ‘increased fluids’ had in fact decreased in some countries. Adding to the concern that these data generate is the fact that earlier studies have illustrated the difficulties in preparing Recommended Home Fluids correctly [26]. The household surveys reviewed in this study did not collect data on knowledge on recipes of RHF, nor on the quality of the fluids actually prepared.

The diarrhea case management indicators in LMICs are consistent with previous studies which have shown that progress in the use of ORT has been slow after 1990 [18, 27]. After IMCI was implemented phase-wise from 1996 till early 2000s in over 100 LMICs [28, 29] diarrhea case management indicators were expected to improve as IMCI also emphasizes on improving healthcare seeking behavior [10]. After IMCI implementation, some countries have experienced a substantial progress in diarrhea management not only in the 1990s [6] but also in the 2000s demonstrating that high utilization rates of ORT are possible [18, 30]. Bangladesh is a notable example for making positive strides in diarrhea case management by reaching up to 80% in ORT use rate and this has been attributed to the commitment shown by various stake holders [31]. Country-level progress in diarrhea case management will help identify reasons for the bottlenecks in low performing countries and learn from strategies and policies of high performing.

An intriguing question that arises is “Why has there not been significant improvement in diarrhea case management?” despite the significant efforts put on diarrhea control program [8] through various approaches [10] and platforms [13] for more than three decades. Some of the explanatory factors being discussed are inefficient communication strategies to the caregivers, knowledge-action gap for giving ORT, [32] inadequate education and understanding of the caregivers about the childhood illnesses [33] and competing priorities of the caregivers in poor households when children fall sick [19]. Stallings has argued that taking a child to a HCP may affect fluid and food intake negatively during episodes of diarrhea, as the HCP may not promote ‘increased fluids’ and ‘continued feeding’ but rather treat the illness with pills, syrups, injections or intravenous fluids [25, 32]. Over-prescription of drugs in diarrhea case management have been documented earlier [34]. DHS-based reports from sub-Saharan Africa and India have shown that private HCPs were less likely to provide ORT and more likely to provide other treatments than the public HCPs [35, 36] and in-depth interviews of Indian HCPs have suggested that a lack of direct dispensing of ORS in the private sector is a major barrier to its use [36]. A rather surprising finding is the declining trend of ‘increased fluids’. This finding could be attributed to the validity of the indicator itself (mothers recall use of ORS better than increased fluid intake) or it may reflect decreased intensity in communicating the message on the importance of giving a child with diarrhoea more fluids to drink than usual.

Continued low use rates for ORS/RHF in diarrhea case management may reflect that activities targeted at an entire population can only reach a certain level of success after which further intervention efforts will need to focus on high-risk groups, such as poor and less educated persons [37]. Diarrhea incidence is associated with lower socio-economic status whereas ORT use has been correlated with higher socio-economic status [38]. Experts have also argued that replacement of disease-specific public health programs, such as diarrheal disease control by more integrated approaches such as IMCI may have negatively affected individual component of IMCI [28]. Lastly, weak health systems and poor access to healthcare in resource-limited settings may be central impediments to scaling up the interventions to improve diarrhea case management irrespective of the program approach adopted [17]. More resources, including well-trained HCPs and community health workers, may be essential elements in advancing diarrhea case management further [39]. Collaborative efforts have been undertaken by the WHO and UNICEF to identify barriers to progress in diarrhoea management and the organisations have jointly suggested solutions for improving case management of childhood diarrhea [40]. Applying an equity lens and socially inclusive policies in child health programs may be essential for making further progress [41].

The DHS and MICS surveys are in most instances the best, and often the only, available sources of national-level data in low- income countries. They provide a unique and historic opportunity for cross-country and time-trend analysis. Still, our results should be interpreted with some caution as household survey data may be affected by various sources of errors, among them recall and reporting biases [42]. Such biases need not be systematic, however, in which case they will have minor influence on the conclusions here.

A potential limitation of this study is that the included survey data only provides information on the children who are alive [30]. This may not be a factor of signiificant importance but if there were children in the households who had died from diarrhoea they would have been more likely to have received poor treatment than not. Hence, not including them is more likely to have led to an overestimation rather than an underestimation of proper case management practices. We could not test socio-economic or geographic differentials in 2-week prevalence of diarrhea and case management indicators since the data analysed were aggregated. However, association of diarrhea with lower socio-economic status has been illustrated in previous reports [35, 38] and geographic variations within countries are well documented in DHS reports [32]. The validity of the indicators that were carefully selected by the WHO and UNICEF after field studies and technical consultations, has been questioned. We believe that validity of the indicators may vary. For instance, recalling the actual amount of fluid intake should be more difficult than recalling the type of treatment received [43]. Nevertheless, our report is comparable to previous studies and also updated data, improved the methods and made a cross-country comparison of indicators [14, 18]. Data on continued feeding during diarrhea and zinc treatment for diarrhea was not included in our analyses since these indicators were only available from 90 DHS but not in MICS and we included the comparable data available from both the surveys to obtain greater coverage to improve generazability of our findings. We hope that future studies of this type will be in a better position to assess the use of this important treatment as more data becomes available from the global surveys.

## Conclusion

Our analyses reiterates that diarrhea case management has progressed very little even a decade after implementation of the IMCI strategy during the late 1990s [29]. Caregivers’ knowledge about diarrhea and their case management practices must be improved. A better understanding of reasons for poor caregivers’ healthcare seeking behavior during episodes of diarrhea is needed. Further, more resources should be allocated to health systems to meet the challenges of diarrhea in poverty-stricken populations, most particularly in low-income countries.

## Abbreviations

CCM: Community case management
DHS: Demographic and health surveys
GAPPD: Global action plan for pneumonia and diarrhea
HCP: Healthcare providers
IF: Increased fluids
IMCI: Management of childhood illness strategy
LMIC: Low-and middle-income countries
MDG: Millennium development goal
MICS: Multiple indicator cluster surveys
RHF: Recommended home fluids
ORT: Oral rehydration therapy
ORS: Oral rehydration salts
WHO: World health organization
UNICEF: United nations children’s fund
